# 3′-(4-Meth­oxy­phen­yl)-4′-phenyl-3*H*,4′*H*-spiro­[1-benzothio­phene-2,5′-isoxazol]-3-one

**DOI:** 10.1107/S1600536811024408

**Published:** 2011-06-30

**Authors:** Adil Boughaleb, Hafid Zouihri, Said Gmouh, Abdelali Kerbal, Mohamed El yazidi

**Affiliations:** a Département de Chimie, Faculté des Sciences, Dhar Mehraz, BP 1796 Atlas, 30000 Fés, Morocco; b Laboratoires de Diffraction des Rayons X, Centre Nationale pour la Recherche Scientifique et Technique, Rabat, Morocco; c Centre Nationale pour la Recherche, Scientifique et Technique, Rabat, Morocco

## Abstract

In the title compound, C_23_H_17_NO_3_S, the thio­phene and isoxazole rings each have an envelope conformation with the spiro C atom linking them forming the flap of the envelope in each case. The dihedral angle between the mean planes of the benzothio­phene ring and isoxazole rings is 81.35 (7)°. In the crystal, an inter­molecular C—H⋯O hydrogen bond links the mol­ecules into a chain running parallel to the *a* axis.

## Related literature

For general background to dipolar-1,3 cyclo­addition reactions, see: Al Houari *et al.* (2010 ▶); Toth *et al.* (1999 ▶); El yazidi *et al.* (1994 ▶). For graph-set analysis, see: Bernstein *et al.* (1995 ▶).
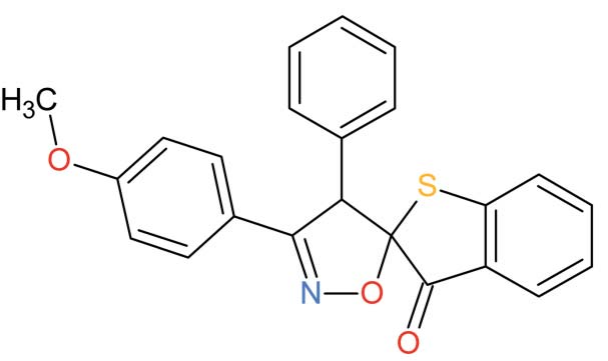


## Experimental

### 

#### Crystal data

C_23_H_17_NO_3_S
*M* *_r_* = 387.44
Triclinic, 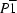

*a* = 9.3644 (13) Å
*b* = 9.8132 (14) Å
*c* = 11.1502 (15) Å
α = 103.575 (8)°
β = 90.360 (8)°
γ = 106.089 (8)°
*V* = 954.2 (2) Å^3^
*Z* = 2
Mo *K*α radiation
μ = 0.19 mm^−1^
*T* = 296 K
0.24 × 0.22 × 0.16 mm

#### Data collection

Bruker APEXII CCD detector diffractometer
14395 measured reflections
4336 independent reflections
3389 reflections with *I* > 2σ(*I*)
*R*_int_ = 0.021

#### Refinement

*R*[*F* ^2^ > 2σ(*F* ^2^)] = 0.040
*wR*(*F* ^2^) = 0.117
*S* = 1.08
4336 reflections
254 parameters
H-atom parameters constrained
Δρ_max_ = 0.30 e Å^−3^
Δρ_min_ = −0.25 e Å^−3^

### 

Data collection: *APEX2* (Bruker, 2005 ▶); cell refinement: *SAINT* (Bruker, 2005 ▶); data reduction: *SAINT*; program(s) used to solve structure: *SHELXS97* (Sheldrick, 2008 ▶); program(s) used to refine structure: *SHELXL97* (Sheldrick, 2008 ▶); molecular graphics: *PLATON* (Spek, 2009 ▶); software used to prepare material for publication: *publCIF* (Westrip, 2010 ▶).

## Supplementary Material

Crystal structure: contains datablock(s) I, global. DOI: 10.1107/S1600536811024408/lw2066sup1.cif

Structure factors: contains datablock(s) I. DOI: 10.1107/S1600536811024408/lw2066Isup2.hkl

Supplementary material file. DOI: 10.1107/S1600536811024408/lw2066Isup3.cml

Additional supplementary materials:  crystallographic information; 3D view; checkCIF report

## Figures and Tables

**Table 1:** Hydrogen-bond geometry (Å, °)

*D*—H⋯*A*	*D*—H	H⋯*A*	*D*⋯*A*	*D*—H⋯*A*
C13—H13⋯O1^i^	0.93	2.60	3.345 (2)	138

Symmetry code: (i) 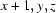.

**Fig. 1.**
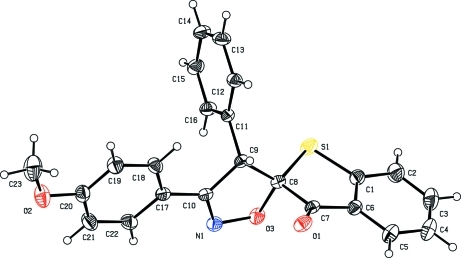
Molecular view of the title compound showing the atom-labeling scheme. Displacement ellipsoids are drawn at the 50% probability level. H atoms are represented as small spheres of arbitrary radii.

**Fig. 2.**
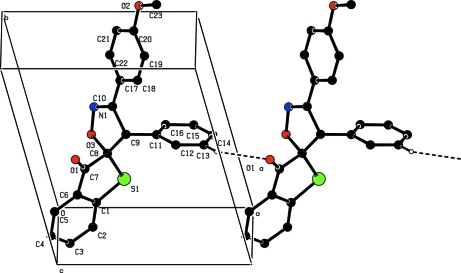
Partial packing view showing the chain formed by C—H···O. H atoms not involved in hydrogen bonds have been omitted for clarity.

## supplementary crystallographic information

### Comment

1,3-dipolar cyclo-addition of arylnitriloxides with ethylenic dipolarophiles produce isoxazolines in which the electron attracting or withdrawing substitutuent of the dipolarophile is at position 5 (IUPAC numbering) of the isoxazoline [Al Houari, *et al.* 2010; Toth *et al.* 1999 and El yazidi *et al.* 1994].

C_23_H_17_NO_3_S, Figure 1, is the product of the reaction of the *p*-anisylnitriloxide with (*Z*)-2-benzylidenebenzo[*b*]thiophen-3(2*H*)- one. The X-ray crystal structure study shows that the hydrogen atom attached to C9 is *cis* to the carbonyl group attached to C7.

The thiophene and isoxazole rings have envelope conformations, the spiro carbon atom linking them forming the flap of the envelope in each case.

The dihedral angles between the of mean planes of the benzothiophene ring, BTh ,(atoms S1 sequentially to C8), the the isoxazole ring, Iso, (atoms N1-O3-C8-C9-C10), the phenyl ring, MPh, (atoms C17 to C22) and the the phenyl ring, Ph, (atoms C11 to C16) are: Bth/Iso = 81.35 (7)°, BTh/MPh = 88.46 (7)°, BTh/Ph = 84.21 (7)°, Iso/MPh = 7.57 (9)°, Iso/Ph = 84.58 (9) and MPh/Ph = 86.41 (9)°.

The C—H···O hydrogen bonds [C13—H13··· O1 (1+*x*, *y*, *z*) (Table 1)] generates C8 chains, (Bernstein *et al.*, 1995), which run parallel to the *a*axis (Figure. 2).

### Experimental

In a 100 ml flask, 2 mmoles of the (*Z*)-2-arylidenebenzo[*b*]thiophen-3(2*H*)-one and 2.2 mmoles of *p*-anisyloxime were dissolved in 20 ml of chloroform. The mixture was cooled to 0°C under magnetic stirring in an ice bath. Then 15 ml of bleach (NaOCl) at 24°Chl(chlorometric degree) was added in small amounts without exceeding the temperature of 5°C. The mixture was left under magnetic stirring for 4 h at room temperature, washed with water until pH was neutral and dried on sodium sulfate. The solvent was evaporated using a rotary evaporator and the oily residue dissolved in ethanol. The resulting precipitate was then re-crystallized in ethanol.

### Refinement

The H atoms bound to C were treated as riding with their parent atoms [C—H distances are 0.93Å for CH groups with *U*_iso_(H) = 1.2 *U*_eq_(C), and 0.97 Å for CH3 groups with *U*_iso_(H) = 1.5 *U*_eq_(C).

### Crystal data

**Table:**

C_23_H_17_NO_3_S	*Z* = 2
*M**_r_* = 387.44	*F*(000) = 404
Triclinic, *P*1	*D*_x_ = 1.348 Mg m^−^^3^
Hall symbol: -P 1	Mo *K*α radiation, λ = 0.71073 Å
*a* = 9.3644 (13) Å	Cell parameters from 312 reflections
*b* = 9.8132 (14) Å	θ = 2.6–26.4°
*c* = 11.1502 (15) Å	µ = 0.19 mm^−^^1^
α = 103.575 (8)°	*T* = 296 K
β = 90.360 (8)°	Prism, colourless
γ = 106.089 (8)°	0.24 × 0.22 × 0.16 mm
*V* = 954.2 (2) Å^3^	

### Data collection

**Table:**

Bruker APEXII CCD detector diffractometer	3389 reflections with *I* > 2σ(*I*)
Radiation source: fine-focus sealed tube	*R*_int_ = 0.021
graphite	θ_max_ = 27.5°, θ_min_ = 2.5°
ω and φ scans	*h* = −12→12
14395 measured reflections	*k* = −12→12
4336 independent reflections	*l* = −14→14

### Refinement

**Table:**

Refinement on *F*^2^	Primary atom site location: structure-invariant direct methods
Least-squares matrix: full	Secondary atom site location: difference Fourier map
*R*[*F*^2^ > 2σ(*F*^2^)] = 0.040	Hydrogen site location: inferred from neighbouring sites
*wR*(*F*^2^) = 0.117	H-atom parameters constrained
*S* = 1.08	*w* = 1/[σ^2^(*F*_o_^2^) + (0.055*P*)^2^ + 0.2963*P*] where *P* = (*F*_o_^2^ + 2*F*_c_^2^)/3
4336 reflections	(Δ/σ)_max_ < 0.001
254 parameters	Δρ_max_ = 0.30 e Å^−^^3^
0 restraints	Δρ_min_ = −0.25 e Å^−^^3^

### Special details

**Table:**

Geometry. All e.s.d.'s (except the e.s.d. in the dihedral angle between two l.s. planes) are estimated using the full covariance matrix. The cell e.s.d.'s are taken into account individually in the estimation of e.s.d.'s in distances, angles and torsion angles; correlations between e.s.d.'s in cell parameters are only used when they are defined by crystal symmetry. An approximate (isotropic) treatment of cell e.s.d.'s is used for estimating e.s.d.'s involving l.s. planes.
Refinement. Refinement of *F*^2^ against ALL reflections. The weighted *R*-factor *wR* and goodness of fit *S* are based on *F*^2^, conventional *R*-factors *R* are based on *F*, with *F* set to zero for negative *F*^2^. The threshold expression of *F*^2^ > σ(*F*^2^) is used only for calculating *R*-factors(gt) *etc*. and is not relevant to the choice of reflections for refinement. *R*-factors based on *F*^2^ are statistically about twice as large as those based on *F*, and *R*- factors based on ALL data will be even larger.

### Fractional atomic coordinates and isotropic or equivalent isotropic displacement parameters (Å^2^)

**Table:**

	*x*	*y*	*z*	*U*_iso_*/*U*_eq_	
S1	0.38947 (5)	0.17699 (5)	0.10614 (4)	0.04579 (15)	
C9	0.48093 (15)	0.45610 (16)	0.28184 (14)	0.0289 (3)	
H9	0.4698	0.4578	0.3695	0.035*	
C11	0.63503 (15)	0.44522 (16)	0.25013 (14)	0.0291 (3)	
C8	0.35039 (16)	0.33637 (17)	0.20326 (14)	0.0311 (3)	
C17	0.53370 (17)	0.74164 (17)	0.31717 (15)	0.0329 (3)	
C7	0.21820 (16)	0.27837 (17)	0.27795 (14)	0.0318 (3)	
C10	0.45054 (16)	0.59076 (17)	0.25599 (14)	0.0309 (3)	
C12	0.72587 (18)	0.41734 (17)	0.33436 (15)	0.0359 (3)	
H12	0.6920	0.4043	0.4103	0.043*	
C16	0.68582 (18)	0.46342 (19)	0.13698 (16)	0.0389 (4)	
H16	0.6253	0.4825	0.0803	0.047*	
C1	0.21339 (18)	0.06249 (18)	0.12568 (16)	0.0387 (4)	
C6	0.13572 (17)	0.12766 (18)	0.21660 (15)	0.0356 (3)	
C18	0.63321 (19)	0.77176 (18)	0.41909 (16)	0.0395 (4)	
H18	0.6484	0.6947	0.4475	0.047*	
C14	0.91775 (19)	0.4278 (2)	0.19263 (18)	0.0458 (4)	
H14	1.0132	0.4233	0.1738	0.055*	
C19	0.7104 (2)	0.91401 (19)	0.47948 (17)	0.0443 (4)	
H19	0.7767	0.9320	0.5477	0.053*	
C22	0.5151 (2)	0.8598 (2)	0.27498 (17)	0.0437 (4)	
H22	0.4502	0.8424	0.2060	0.052*	
C21	0.5915 (2)	1.0009 (2)	0.33447 (19)	0.0498 (5)	
H21	0.5781	1.0782	0.3053	0.060*	
C13	0.86727 (19)	0.4089 (2)	0.30515 (18)	0.0457 (4)	
H13	0.9284	0.3905	0.3617	0.055*	
C20	0.6883 (2)	1.02933 (18)	0.43755 (17)	0.0424 (4)	
C15	0.82683 (19)	0.4533 (2)	0.10813 (18)	0.0442 (4)	
H15	0.8601	0.4637	0.0315	0.053*	
C5	−0.0038 (2)	0.0500 (2)	0.24311 (18)	0.0490 (4)	
H5	−0.0552	0.0939	0.3045	0.059*	
C2	0.1529 (2)	−0.0820 (2)	0.0605 (2)	0.0548 (5)	
H2	0.2046	−0.1271	0.0003	0.066*	
C3	0.0136 (3)	−0.1569 (2)	0.0876 (2)	0.0628 (6)	
H3	−0.0285	−0.2536	0.0442	0.075*	
C4	−0.0650 (2)	−0.0928 (2)	0.1771 (2)	0.0617 (6)	
H4	−0.1589	−0.1457	0.1928	0.074*	
C23	0.8590 (3)	1.2094 (3)	0.5952 (2)	0.0779 (7)	
H23A	0.8091	1.1682	0.6589	0.117*	
H23B	0.8948	1.3139	0.6248	0.117*	
H23C	0.9416	1.1708	0.5735	0.117*	
O1	0.19215 (13)	0.35372 (14)	0.37248 (11)	0.0448 (3)	
O2	0.75789 (18)	1.17292 (14)	0.48941 (14)	0.0628 (4)	
O3	0.28865 (12)	0.40970 (13)	0.12447 (10)	0.0405 (3)	
N1	0.34693 (15)	0.56346 (15)	0.17074 (13)	0.0389 (3)	

### Atomic displacement parameters (Å^2^)

**Table:**

	*U*^11^	*U*^22^	*U*^33^	*U*^12^	*U*^13^	*U*^23^
S1	0.0348 (2)	0.0447 (3)	0.0485 (3)	0.00839 (18)	0.00969 (18)	−0.00299 (19)
C9	0.0243 (7)	0.0343 (7)	0.0270 (7)	0.0066 (6)	0.0000 (5)	0.0077 (6)
C11	0.0248 (7)	0.0287 (7)	0.0324 (8)	0.0065 (6)	−0.0008 (6)	0.0062 (6)
C8	0.0265 (7)	0.0363 (8)	0.0300 (7)	0.0095 (6)	0.0015 (6)	0.0068 (6)
C17	0.0298 (7)	0.0372 (8)	0.0339 (8)	0.0119 (6)	0.0038 (6)	0.0102 (6)
C7	0.0239 (7)	0.0397 (8)	0.0324 (8)	0.0087 (6)	−0.0005 (6)	0.0104 (6)
C10	0.0251 (7)	0.0380 (8)	0.0315 (8)	0.0110 (6)	0.0031 (6)	0.0101 (6)
C12	0.0358 (8)	0.0379 (8)	0.0358 (8)	0.0132 (7)	−0.0012 (6)	0.0096 (7)
C16	0.0319 (8)	0.0512 (10)	0.0393 (9)	0.0150 (7)	0.0033 (7)	0.0185 (8)
C1	0.0359 (8)	0.0380 (8)	0.0394 (9)	0.0075 (7)	−0.0017 (7)	0.0077 (7)
C6	0.0319 (8)	0.0387 (8)	0.0350 (8)	0.0065 (6)	0.0001 (6)	0.0109 (7)
C18	0.0442 (9)	0.0353 (8)	0.0398 (9)	0.0119 (7)	−0.0041 (7)	0.0107 (7)
C14	0.0287 (8)	0.0481 (10)	0.0607 (12)	0.0151 (7)	0.0047 (8)	0.0087 (9)
C19	0.0482 (10)	0.0418 (9)	0.0392 (9)	0.0088 (8)	−0.0066 (8)	0.0077 (7)
C22	0.0437 (9)	0.0457 (10)	0.0456 (10)	0.0155 (8)	−0.0049 (8)	0.0155 (8)
C21	0.0590 (11)	0.0386 (9)	0.0568 (12)	0.0165 (8)	−0.0005 (9)	0.0187 (8)
C13	0.0376 (9)	0.0507 (10)	0.0528 (11)	0.0209 (8)	−0.0076 (8)	0.0109 (8)
C20	0.0453 (9)	0.0343 (8)	0.0446 (10)	0.0085 (7)	0.0072 (8)	0.0074 (7)
C15	0.0358 (9)	0.0522 (10)	0.0475 (10)	0.0138 (8)	0.0133 (7)	0.0164 (8)
C5	0.0388 (9)	0.0551 (11)	0.0472 (10)	0.0018 (8)	0.0070 (8)	0.0147 (9)
C2	0.0566 (12)	0.0413 (10)	0.0575 (12)	0.0094 (9)	0.0010 (9)	0.0005 (9)
C3	0.0654 (13)	0.0394 (10)	0.0675 (14)	−0.0046 (9)	−0.0059 (11)	0.0061 (9)
C4	0.0480 (11)	0.0546 (12)	0.0682 (14)	−0.0106 (9)	0.0027 (10)	0.0180 (11)
C23	0.0882 (18)	0.0500 (12)	0.0695 (16)	−0.0054 (12)	−0.0139 (13)	−0.0046 (11)
O1	0.0353 (6)	0.0540 (7)	0.0383 (6)	0.0110 (5)	0.0076 (5)	0.0005 (6)
O2	0.0791 (10)	0.0359 (7)	0.0628 (9)	0.0036 (7)	−0.0049 (8)	0.0074 (6)
O3	0.0368 (6)	0.0441 (6)	0.0361 (6)	0.0029 (5)	−0.0108 (5)	0.0124 (5)
N1	0.0353 (7)	0.0421 (8)	0.0398 (8)	0.0098 (6)	−0.0037 (6)	0.0128 (6)

### Geometric parameters (Å, °)

**Table:**

S1—C1	1.7667 (18)	C15—C16	1.386 (3)
S1—C8	1.8111 (17)	C17—C18	1.387 (2)
O1—C7	1.206 (2)	C17—C22	1.401 (3)
O2—C20	1.360 (2)	C18—C19	1.385 (3)
O2—C23	1.420 (3)	C19—C20	1.385 (3)
O3—N1	1.4196 (19)	C20—C21	1.385 (3)
O3—C8	1.465 (2)	C21—C22	1.372 (3)
N1—C10	1.281 (2)	C2—H2	0.9300
C1—C2	1.388 (3)	C3—H3	0.9300
C1—C6	1.389 (2)	C4—H4	0.9300
C2—C3	1.382 (3)	C5—H5	0.9300
C3—C4	1.380 (3)	C9—H9	0.9800
C4—C5	1.378 (3)	C12—H12	0.9300
C5—C6	1.391 (3)	C13—H13	0.9300
C6—C7	1.462 (2)	C14—H14	0.9300
C7—C8	1.548 (2)	C15—H15	0.9300
C8—C9	1.533 (2)	C16—H16	0.9300
C9—C10	1.515 (2)	C18—H18	0.9300
C9—C11	1.514 (2)	C19—H19	0.9300
C10—C17	1.464 (2)	C21—H21	0.9300
C11—C12	1.386 (2)	C22—H22	0.9300
C11—C16	1.384 (2)	C23—H23A	0.9600
C12—C13	1.386 (3)	C23—H23B	0.9600
C13—C14	1.378 (3)	C23—H23C	0.9600
C14—C15	1.376 (3)		
			
C1—S1—C8	91.79 (8)	C18—C19—C20	119.55 (17)
C20—O2—C23	118.54 (17)	O2—C20—C19	124.60 (17)
N1—O3—C8	109.06 (11)	O2—C20—C21	115.76 (16)
O3—N1—C10	109.34 (13)	C19—C20—C21	119.62 (17)
S1—C1—C2	125.36 (14)	C20—C21—C22	120.64 (18)
S1—C1—C6	114.40 (13)	C17—C22—C21	120.68 (17)
C2—C1—C6	120.23 (17)	C1—C2—H2	121.00
C1—C2—C3	118.11 (19)	C3—C2—H2	121.00
C2—C3—C4	122.11 (19)	C2—C3—H3	119.00
C3—C4—C5	119.72 (19)	C4—C3—H3	119.00
C4—C5—C6	119.07 (18)	C3—C4—H4	120.00
C1—C6—C5	120.74 (16)	C5—C4—H4	120.00
C1—C6—C7	112.67 (15)	C4—C5—H5	120.00
C5—C6—C7	126.58 (16)	C6—C5—H5	120.00
O1—C7—C6	127.74 (15)	C8—C9—H9	109.00
O1—C7—C8	121.67 (15)	C10—C9—H9	109.00
C6—C7—C8	110.59 (13)	C11—C9—H9	109.00
S1—C8—O3	108.71 (10)	C11—C12—H12	120.00
S1—C8—C7	106.43 (11)	C13—C12—H12	120.00
S1—C8—C9	117.97 (11)	C12—C13—H13	120.00
O3—C8—C7	103.57 (12)	C14—C13—H13	120.00
O3—C8—C9	105.03 (12)	C13—C14—H14	120.00
C7—C8—C9	114.10 (12)	C15—C14—H14	120.00
C8—C9—C10	100.13 (12)	C14—C15—H15	120.00
C8—C9—C11	115.78 (13)	C16—C15—H15	120.00
C10—C9—C11	112.61 (13)	C11—C16—H16	120.00
N1—C10—C9	114.45 (14)	C15—C16—H16	120.00
N1—C10—C17	120.87 (15)	C17—C18—H18	119.00
C9—C10—C17	124.63 (14)	C19—C18—H18	119.00
C9—C11—C12	120.14 (14)	C18—C19—H19	120.00
C9—C11—C16	120.12 (14)	C20—C19—H19	120.00
C12—C11—C16	119.75 (15)	C20—C21—H21	120.00
C11—C12—C13	119.81 (16)	C22—C21—H21	120.00
C12—C13—C14	120.29 (17)	C17—C22—H22	120.00
C13—C14—C15	119.98 (17)	C21—C22—H22	120.00
C14—C15—C16	120.16 (18)	O2—C23—H23A	109.00
C11—C16—C15	120.01 (16)	O2—C23—H23B	110.00
C10—C17—C18	120.82 (15)	O2—C23—H23C	109.00
C10—C17—C22	121.19 (15)	H23A—C23—H23B	109.00
C18—C17—C22	117.99 (16)	H23A—C23—H23C	109.00
C17—C18—C19	121.50 (16)	H23B—C23—H23C	109.00

### Hydrogen-bond geometry (Å, °)

**Table:**

*D*—H···*A*	*D*—H	H···*A*	*D*···*A*	*D*—H···*A*
C13—H13···O1^i^	0.93	2.60	3.345 (2)	138

Symmetry codes: (i) *x*+1, *y*, *z*.
